# Atypical Presentation of Papillon–Lefèvre Syndrome: A Case of Isolated Cutaneous Manifestations Without Dental Involvement

**DOI:** 10.3390/reports8040190

**Published:** 2025-09-26

**Authors:** Mishari Alrubaiaan, Mansour Almutairi, Waleed Alajroush

**Affiliations:** 1 College of Medicine, King Saud Bin Abdulaziz University for Health Sciences, Riyadh 11481, Saudi Arabia; 2 Dermatology Department, King Abdulaziz Medical City, Riyadh 11426, Saudi Arabia; 3 Department of Pediatric Dermatology, King Abdulaziz Medical City, Riyadh 11426, Saudi Arabia

**Keywords:** PLS, keratoderma, Pediatrics

## Abstract

**Background and Clinical Significance:** Papillon–Lefèvre syndrome (PLS) is an autosomal recessive genetic skin disorder. Genetic studies have demonstrated that mutations in the Cathepsin-C (CTSC) gene, mapped to chromosome 11q14.1–q14.3, are responsible for the pathogenesis of PLS. The hallmark characteristics of this syndrome are palmoplantar keratoderma and severe periodontal disease that leads to premature tooth loss. Palmoplantar keratoderma commonly manifests during early childhood (ages one to four), followed by the onset of severe periodontitis around the age of three to four years. Although periodontitis and premature tooth loss are considered hallmark features, a limited number of cases lacking oral involvement have been reported, underscoring the phenotypic variability in PLS. **Case Presentation:** This report describes a 6-year-old female patient whose chief presenting complaint was palmoplantar keratoderma, recurrent skin infections, necrotizing granulomatous inflammation of the kidney, and delayed growth; she was genetically confirmed to have a CTSC mutation associated with PLS, yet without any dental manifestations. The lack of oral manifestations and the presence of necrotizing granulomatous inflammation of the kidney in this genetically validated case highlight an atypical presentation. **Conclusions:** This report discusses an unusual case of PLS of a patient displaying classic skin features without any dental issues.

## 1. Introduction and Clinical Significance

Palmoplantar keratoderma, which encompasses a group of conditions, is marked by the skin on the palms and soles becoming excessively thick [1]. These conditions can be acquired, inherited, or linked to other syndromes, such as PLS [2]. In 1924, Papillon and Lefèvre released the first report on PLS [3]. They described two siblings who experienced premature tooth loss, periodontitis that began in early childhood, and palmoplantar hyperkeratosis [3]. The primary abnormality in PLS is the CTSC gene, which codes for the dipeptidyl aminopeptidase enzyme cathepsin C [4]. Symptoms associated with this condition include dura mater calcifications, early dental loss, and palmoplantar hyperkeratosis [5]. Characteristic findings include sharply demarcated, erythematous keratotic plaques affecting the palmar and plantar surfaces, with some cases demonstrating spread onto the dorsal regions of the hands and feet [1]. Skin lesions are most frequently observed in early childhood, usually prior to the age of four [5]. The second hallmark of PLS is severe periodontitis, which typically begins at around three to four years of age [1]. Although the eruption of deciduous teeth is normal, it is accompanied by significant gingival inflammation, marked by redness, swelling, and a propensity to bleed easily [1]. By the time a child is four years old, most of their primary teeth have fallen out. After that, the gums look healthy again [1]. As the permanent teeth erupt, the characteristic cycle of gingival inflammation and destructive periodontitis resumes, culminating in the premature exfoliation of the permanent dentition [1].

In addition to these features, palmoplantar keratoderma, psoriasiform lesions, and recurrent skin infections occur more frequently than liver abscesses, hyperhidrosis, dural calcifications, and intellectual disability [1,5]. PLS is exceedingly rare, with an estimated prevalence of 1–4 cases per million individuals, making the recognition and reporting of atypical variants clinically significant [5]. This condition affects both genders similarly, and there is no racial preference [1]. The disorder follows an autosomal recessive pattern of inheritance, with consanguinity frequently observed among affected individuals [2]. Consanguinity is noted in 20–40% of PLS cases [1]. Multiple factors contribute to the development of PLS, including microbiological, immunological, and genetic elements [5].

There is a broad spectrum of phenotypic expression resulting from CTSC mutations, but severe periodontal disease remains a defining characteristic [4]. Only a few genetically confirmed cases without oral manifestations have been described, underscoring phenotypic variability and providing context for the rarity of our patient’s presentation [6,7]. Herein, we report a 6-year-old girl presenting with palmoplantar keratoderma, recurrent skin infections, necrotizing granulomatous inflammation, and delayed growth, who was genetically verified to have a CTSC mutation linked to PLS, despite the absence of oral manifestations. The absence of oral symptoms in this genetically confirmed case underscores its unusual presentation.

## 2. Case Presentation

A 6-year-old girl attended our pediatric dermatology clinic due to erythema, hyperkeratosis, and hyperhidrosis affecting her hands and feet. She was delivered via an uncomplicated pregnancy and showed normal growth and development in her early childhood. Neither of her parents was consanguineous nor had they suffered from similar health issues. In addition, neither of her siblings was affected by the same issue. Alongside her skin problems, she had recurrent high fevers, cutaneous infections, and episodes of diarrhea occurring every two to three weeks starting at six months of age.

At the age of four, she was referred to the pediatric endocrinology department due to her short stature. Using Greulich and Pyle’s Atlas, doctors found a delay of two years in her bone age, suggesting a constitutional growth delay. By the time she was five, a pediatric hematologist, gastroenterologist, and infectious disease specialist evaluated her for her anemia, leukocytosis, and increased inflammatory markers. Imaging studies revealed hepatic lesions and a left retroperitoneal and renal mass. A renal biopsy indicated she had necrotizing granulomatous inflammation, along with chronic disease-related iron deficiency anemia.

**Figure 1:**
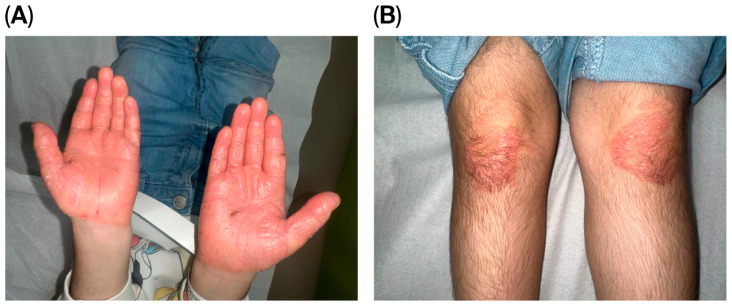
(**A**) Bilateral erythematous, thick, hyperkeratotic lesions with overlying scales over the hands and (**B**) multiple well-defined, irregular erythematous hyperkeratotic plaques over the plantar surface of the hand and extensor surfaces of the knees.

**Figure 2:**
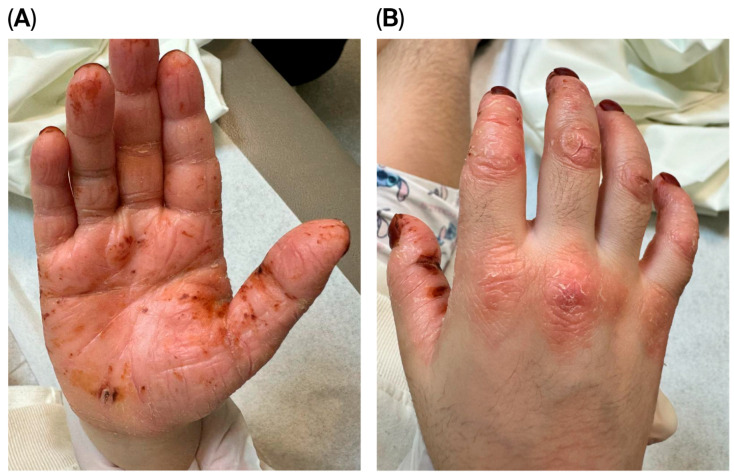
(**A**) Thickened, hyperkeratotic skin with multiple erosions, fissures, and hemorrhagic crusts. (**B**) Erythematous hyperkeratotic plaques with overlying scales over the knuckles.

A thorough examination showed her to be thin and shorter than average, consistent with failure to thrive. Despite this, she walked steadily and displayed no signs of cognitive impairment. The skin assessment revealed palmoplantar keratoderma and well-defined, psoriatic-like erythematous keratotic plaques bilaterally affecting her palms, soles, and knees Figure 1 and Figure 2. She had no features of ichthyosis, erythroderma, or generalized scaling. In addition, she had no hair or nail abnormalities. An oral examination found no gingival inflammation, generalized periodontitis, deep pocketing around teeth, hypermobile molars, or halitosis.

Based on her clinical findings and the lack of oral manifestations, we initially diagnosed her with pityriasis rubra pilaris. However, whole-genome sequencing was performed and verified that she has Papillon–Lefèvre syndrome resulting from a homozygous CTSC c.815G>C, p.(Arg272Pro) variant. According to the Medical Genetics and Genomics (ACMG) guidelines, this variant has been reported as pathogenic (ClinVar Variation ID: 548504). Analysis of her parents and brother revealed that they were heterozygous for this mutation.

In order to assess and evaluate her suitability for biologic therapy, laboratory screening tests were conducted. We tested her for tuberculosis as well as for hepatitis, and the results came back negative. The patient was put on adalimumab 20 mg every two weeks. This was done in conjunction with the consistent application of topical moisturizers. Since the beginning of treatment, the patient has been monitored on a monthly basis, and it has been observed that her keratosis has been steadily improving. Additionally, the patient’s oral intake and physical activity have also significantly improved. The patient’s clinical, laboratory, and genetic findings are summarized in Table 1.

## 3. Discussion

In 1924, Papillon and Lefèvre made a discovery pertaining to PLS [3]. It was determined later that PLS is inherited in an autosomal recessive manner within families [1,2]. It impacts individuals of all genders uniformly [1]. Moreover, it is also observed that it tends to show no racial inclination [1]. It is notably rare, with an incidence of 1 to 4 instances per million [5]. PLS often manifests only when a child receives the gene from both parents, despite the parents generally exhibiting no symptoms [5].

PLS is usually caused by mutations in the cathepsin C gene, which encodes a lysosomal protease located on chromosome 11q14–q21 [5]. The CTSC gene produces a cysteine lysosomal protease, known as dipeptidyl-peptidase I, which works by removing dipeptides from the amino ends of protein molecules [8]. This gene is highly active in epithelial tissues and in immune cells. There seem to be two primary elements involved in the development of PLS. Some individuals have compromised cellular immunity, as seen by decreased mobility and pathogen-engulfing capacity of neutrophils and granulocytes. In some cases, the illness appears to be fueled by periodontal infections brought on by bacteria such as spirochetes and Peptostreptococcus micros. In addition to this, other bacteria are also considered, such as Fusobacterium nucleatum, Porphyromonas gingivalis, and Actinobacillus actinomycetemcomitans. [1,5].

Palmoplantar keratoderma (PPK) affecting the hands and feet is a common manifestation of PLS [5]. During the winter months, palmoplantar keratoderma frequently becomes worse [1]. It causes painful fissures that hinder ambulation and make it difficult for the patient to use their hands [1]. By the time the child is three or four years old, they usually have rapid progressive periodontitis and loss of alveolar bone [5]. In this instance, pityriasis rubra pilaris was first considered as a potential diagnosis because the patient lacked oral symptoms. The homozygous CTSC c.815G>C, p.(Arg272Pro) mutation identified in our patient has previously been reported in several cases of PLS. Palmoplantar keratoderma and early onset of oral manifestations are also usually associated with this variant [9]. The absence of oral manifestations in our patient, who had the same mutation, further emphasizes the diversity of PLS presentation. Similarly, the diversity of PLS presentations has been reported by Abderl-Hamid et al. (2024) [10]. Their analysis of twelve Egyptian patients with PLS showed 5 distinct CTSC mutations and a broad spectrum of clinical manifestations. Some cases had severe dental symptoms while others had minor or atypical features [10]. Our case is considered to be very uncommon because PLS often manifests with both cutaneous and oral symptoms. This indicates that clinical variations may undermine the established understanding of PLS.

For example, Ochiai et al. (2009) described a 14-year-old girl who had characteristic palmoplantar keratoderma but who showed no symptoms of early tooth loss or periodontitis [6]. Likewise, an adult patient with no periodontal issues and healthy permanent teeth was reported by Kobayashi et al. (2013) [7]. Although the lack of oral manifestation has been rarely reported, this limited number of reports clearly indicates that PLS may occur without dental symptoms. The precise causes of the absence of oral involvement remain unidentified; however, genetic, developmental, immunological, and environmental factors may contribute. The residual neutrophil serine protease activity may play a role in maintaining host defense at the gingival level, thereby preventing periodontal breakdown [11]. In addition, the different pathogens colonizing the gingiva, such as Aggregatibacter actinomycetemcomitans, may have a protective impact by hindering the inflammatory cascade observed in PLS [5]. By presenting a genetically verified child with typical skin characteristics and a lack of oral manifestations, our case adds to this limited body of knowledge. In addition, it points out how PLS may show up in a variety of ways and stresses how important it is for physicians to recognize and understand these unusual presentations. Furthermore, unlike systemically healthy individuals, our case displayed recurrent infections, necrotizing granulomatous inflammation, and growth delay, pointing toward systemic immune dysregulation [6,7].

It is becoming well known that systemic infections are part of the clinical spectrum of PLS. Recurrent infections of the skin are also extremely common in PLS, affecting 20% of individuals [8,12,13]. It is important to note that Pyogenic liver abscesses have also recently been reported in PLS patients. Almuneef et al. (2003) documented four pediatric instances and reported that pyogenic abscesses are more common and frequent than previously believed [8]. They are also considered to be a result of weakened neutrophil-mediated host defenses [8]. Similarly, Basu et al. (2022) described two children who experienced recurrent liver abscesses and required surgical drainage, corticosteroids, as well as long-term antibiotics to control excessive inflammatory responses [14]. On the other hand, instead of developing a pyogenic liver abscess, our patient had necrotizing granulomatous inflammation of the kidney, which further enhances the known systemic manifestations of PLS. Abnormal granulomatous inflammatory pathways may be involved in the immunological dysregulation of PLS. Furthermore, our patient’s delayed growth and recurrent skin infections raise the possibility that PLS might have a systemic impact as well.

In order to improve PLS outcomes, one of the main aims should be early detection. Even though periodontitis is a defining characteristic, the diagnosis should always be suspected when a child exhibits systemic inflammatory symptoms, skin infections, and early-onset palmoplantar keratoderma. To distinguish it from other hereditary keratodermas, however, genetic evidence of a CTSC mutation becomes crucial in such unusual instances with no oral involvement.

PLS should always be treated in a multidisciplinary approach. Strong collaboration between pediatricians, as well as pediatric dentists and dermatologists, is necessary [15]. For PPK, topical therapies such as emollients combined with urea, salicylic acid, or retinoids are most commonly used [16]. It is very important to note that our patient’s palmoplantar keratoderma improved significantlyalong with physical activity as well as oral intake after receiving biologics treatment with adalimumab. While biologics have been used in isolated cases of palmoplantar keratoderma, their role in PLS remains largely unexplored and warrants further investigation. A recent report revealed a remarkable clinical response to adalimumab in a patient with PLS, implying that TNF-induced inflammation may contribute to disease activity and that inhibiting TNF may provide a therapeutic benefit in PLS patients [17].

Effective management of periodontal disease in PLS generally consists of prompt antibiotic therapy combined with nonsurgical approaches, reinforcement of oral hygiene, extraction of primary teeth, and regular follow-up [16].

## 4. Conclusions

Papillon–Lefèvre syndrome has a profound impact on psychological, social, and aesthetic well-being from an early age, as the disease is characterized by skin involvement and partial or complete loss of teeth. We highlight the occurrence of only cutaneous symptoms without any dental structure involvement, underscoring the critical need to recognize atypical presentations and maintain clinical awareness regarding phenotypic variability in diagnosing and managing PLS. Moreover, the presence of necrotizing granulomatous inflammation involving the kidney in our patient represents an additional unique systemic manifestation, broadening the recognized spectrum of this disorder.

## Figures and Tables

**Table 1:** Clinical and laboratory findings in our patient with Papillon–Lefèvre syndrome.

Domain	Finding
Demographics	Six-year-old girl
Genetics	Homozygous CTSC c.815G>C, p.(Arg272Pro) variant (confirmed by genetic testing); classified as pathogenic per ACMG guidelines; parents and brother heterozygous carriers
Dermatologic features	Palmoplantar keratoderma with erythema and hyperkeratosis; hyperhidrosis of hands and feet; psoriatic-like erythematous keratotic plaques on palms, soles, and knees
Dental/oral findings	Normal dentition; no gingival inflammation, no periodontitis, no deep pockets, no hypermobile molars, no halitosis
Systemic manifestations	Recurrent high fevers; recurrent cutaneous infections; episodes of diarrhea since 6 months of age; necrotizing granulomatous inflammation of the kidney (renal biopsy) and hepatic lesions; failure to thrive; growth delay (bone age delayed by 2 years)
Laboratory tests	Anemia (chronic disease-related iron deficiency); leukocytosis; increased inflammatory markers; hepatitis and tuberculosis screening negative
Histopathology	Renal biopsy: necrotizing granulomatous inflammation
Treatment and response	Adalimumab 20 mg every 2 weeks + topical emollients; monthly follow-up showed gradual improvement in keratoderma, physical activity, and oral intake
Inheritance background	Non-consanguineous parents: autosomal recessive inheritance confirmed

## Data Availability

The original data presented in the study are included in the article, further inquiries can be directed to the corresponding author.

## Abbreviations

The following abbreviations are used in this manuscript:

PLS	Papillon–Lefèvre syndrome
